# Genetic variation and factors affecting the genetic structure of the lichenicolous fungus *Heterocephalacria bachmannii* (Filobasidiales, Basidiomycota)

**DOI:** 10.1371/journal.pone.0189603

**Published:** 2017-12-18

**Authors:** Raquel Pino-Bodas, Into Laakso, Soili Stenroos

**Affiliations:** 1 Real Jardín Botánico de Madrid, CSIC, Madrid, Spain; 2 Division of Pharmaceutical Biosciences, Faculty of Pharmacy, University of Helsinki, Helsinki, Finland; 3 Botanical Museum, Finnish Museum of Natural History, University of Helsinki, Helsinki, Finland; Ruhr-Universitat Bochum, GERMANY

## Abstract

*Heterocephalacria bachmannii* is a lichenicolous fungus that takes as hosts numerous lichen species of the genus *Cladonia*. In the present study we analyze whether the geographical distance, the host species or the host secondary metabolites determine the genetic structure of this parasite. To address the question, populations mainly from the Southern Europe, Southern Finland and the Azores were sampled. The specimens were collected from 20 different host species representing ten chemotypes. Three loci, ITS rDNA, LSU rDNA and mtSSU, were sequenced. The genetic structure was assessed by AMOVA, redundance analyses and Bayesian clustering methods. The results indicated that the host species and the host secondary metabolites are the most influential factors over the genetic structure of this lichenicolous fungus. In addition, the genetic structure of *H*. *bachmannii* was compared with that of one of its hosts, *Cladonia rangiformis*. The population structure of parasite and host were discordant. The contents in phenolic compounds and fatty acids of *C*. *rangiformis* were quantified in order to test whether it had some influence on the genetic structure of the species. But no correlation was found with the genetic clusters of *H*. *bachmannii*.

## Introduction

The term “lichenicolous fungi” refers to a polyphyletic group of fungi specialized in living on lichens, whether as parasites, as commensals, or as saprophytes [1–2]. It is widely accepted that most of the species of lichenicolous fungi are highly specific, restricting themselves to a few host species that generally belong to a single genus [2–3]. Nevertheless, little is known about the factors that determine the host specificity and the genetic structure of the lichenicolous fungi. Very few researches using molecular tools have addressed these questions thus far. Molina et al. [4] studied the genetic variation of the lichenicolous basidiomyceteous fungus *Marchandiomyces corallinus* (Roberge) Diederich & D. Hawksw., that grows on host species of different lichen genera; they found that the genetic variation of this species was geographically structured. Werth et al. [5] studied the populations of *Tremella lobariacearum* Diederich & M.S. Christ. in Macaronesia on three host species of the genus *Lobaria*. In this case the authors found that the host species was the most important factor in the genetic structure of *T*. *lobariacearum*. Millanes et al. [6] investigated the genetic variation of *Biatoropsis usnearum* Räsänen, finding that it constitutes a complex of cryptic species, and that the host specificity seems to have influenced the speciation. Nadler [7] proposed that the host species can be the most determining factor for the genetic structure of the parasites. However, other possible factors, like the secondary metabolites of the host, could have a similar relevance in the lichenicolous fungi. Lichens synthesize a great variety of secondary metabolites, which are chemically variable and exclusive to them. These metabolites belong to aliphatic acids, lactones, quinones, dibenzofuranes, depsides, depsidones, terpenoids, xanthones, steroids, and carotenoids [8–9]. One of the biological functions attributed to secondary metabolites in the lichen hosts are the protection against the parasites [2, 10, 11], therefore they could play an important role in the genetic structure of lichenicolous fungi. It has been suggested that the different species of lichenicolus fungi are tolerant to only a limited number of these substances, and would select their hosts according to the substances they produce [12–13]. A different tolerance degree to several lichen compounds has been proved in fungi with a lichenicolous life style [14]; therefore, the secondary metabolites of the host could be a relevant factor of adaptive divergence for these organisms. To date, however, no research has been carried out in order to test whether any connection exists between the genetic variation of the lichenicolous fungi and the secondary metabolites of the host.

The genus *Heterocephalacria* (Filobasidiales, Basidiomycota) includes four species of gelatinous fungi, all of them mycoparasites, two of which are lichenicolous [15–16]. *Heterocephalacria bachmannii* (Diederich & M.S. Christ.) Millanes & Wedin is a widely distributed species, reported from Europe, Macaronesia, North America, and Asia [17–18]. This fungus induces the formation of gelatinous, red-brownish, more or less elongated galls that often cause deformations in the host thallus, but never kill it. Little is known of the life cycle of *H*. *bachmannii*–e.g. its mating system (homothallic or heterothallic) or dispersal mechanisms. This species parasitizes only species of the genus *Cladonia* [17] and it has been found so far on 39 species either on primary thalli or on podetia [18].

Most of the species of the genus *Cladonia* are terricolous, growing in areas of high light radiation and humidity [19]. The species that are hosts for *H*. *bachmannii* synthesize a great variety of secondary metabolites. These include aliphatic acids such as rangiformic and bourgeanic acids; depsides such as barbatic acid, depsidones such as fumarprotocetraric acid, the dibenzofuran usnic acid and terpenoid such as zeorin. In addition, a great number of species of *Cladonia* are chemically very variable [19–20], i.e. different specimens of the same species can synthesize different secondary metabolites. Though most of *Cladonia* species can reproduce sexually by means of apothecia, the majority of the species frequently lack apothecia, implying that asexual reproduction is predominant by dispersion of vegetative propagules, such as soredia, granules, squamules or thallus fragments [19]. Therefore, *H*. *bachmannii* could codisperse along with the host by these fragments carrying fungus galls; in this case we would expect a high congruence between the genetic structures of the parasite and its host. The system *Cladonia–H*. *bachmannii* is therefore suitable to study which of the agents determine the genetic structure of the lichenicolous fungi.

The aim of this study was to examine the genetic structure of *H*. *bachmannii* and the factors that determine it, assessing the importance of three potential factors: host species, host secondary metabolites, and geographical distribution.

## Material and methods

### Sampling and loci selection

The specimens were collected between July 2014 and July 2015 in areas belonging to three different biogeographical regions: the Azores Islands (Macaronesian region), Southern Europe (Mediterranean region) and Southern Finland (Hemiboreal region). On every locality, one to five specimens of *H*. *bachmannii* per host species were collected. The collected specimens were spaced at least 5 m from each other. To complete the sampling, specimens deposited in the herbaria H, LE, and MACB were selected, including eight specimens from America, and two from Asia. The new collections were deposited in H. In total, DNA sequences from 123 specimens of *H*. *bachmannii* were obtained (Table 1). The *Cladonia* host species were identified according to Ahti & Stenroos [20], by morphological and chemical study of the specimens.

**Table 1:** Populations of *H*. *bachmannii* collected on several *Cladonia* host species, with the secondary metabolites detected on the host. The specimens from herbaria are indicated in brackets, N = number of specimens.

Population	Coordinates	Host	Host metabolites	N
Spain, Zaragoza, Vera del Mocayo	41°48'51"N, 1°43'23"W	*C*. *rangiformis*	ATR, RANG, NRANG, FUM, PRO	3
		*C*. *foliacea*	USN, FUM, PRO	2
Spain, Zaragoza, Tarazona	41°49'48"N, 1°48'58"W	*C*. *rangiformis*	ATR, RANG, NRANG	2
Spain, Toledo, Torrico	39°50'35"N, 5°12'36"W	*C*. *rangiformis*	ATR, RANG, NRANG, FUM, PRO	2
Spain, Toledo, Sevilleja de la Jara	39°28'42"N, 5°00'12"W	*C*. *rangiformis*	ATR, RANG, NRANG	2
Spain, Toledo, Navaltoril	39°33'45"N, 4°47'56"W	*C*. *glauca*	SQUAM	3
Spain, Toledo, Las Hunfrías	39°34'45"N, 4°47'56"W	*C*. *rangiformis*	ATR, RANG, NRANG	3
Spain, Toledo, Aldeanueva de Barbarroya	39°42'2"N, 5°04'23"W	*C*. *rangiformis*	ATR, RANG, NRANG	3
		*C*. *furcata*	FUM, PRO	1
Spain, Soria, Matalebrera	41°49'38"N, 2°4'22"W	*C*. *rangiformis*	ATR, RANG, NRANG, FUM, PRO	2
Spain, Soria, Muriel Viejo (MACB)	41°46’45”N, 2°55’13”W	*C*. *uncialis*	USN, SQUAM	2
		*C*. *furcata*	FUM, PRO	1
Spain, Salamanca, La Alberca (MACB)	40°27’N, 6°06’W	*C*. *uncialis*	USN, SQUAM	2
Spain, Cáceres, between Puerto de San Vicente and Alias	39°30'11"N, 5°6'40"W	*C*. *rangiformis*	ATR, RANG, NRANG	1
		*C*. *foliacea*	USN, FUM, PRO	1
Spain, Cáceres, Guadalupe (MACB)	39°26’23”N, 5°25’15”W	*C*. *uncialis* subsp. *biuncialis*	USN, SQUAM	1
Turkey, Çankırı	41°02'48"N, 33° 44'15"E	*C*. *pyxidata*	FUM, PRO	2
Turkey, Kastamonu, Ilgaz Dağı	41°04'45"N, 33°44'07"E	*C*. *coniocraea*	FUM, PRO	1
Turkey, Kastamonu, Pınarbaşı	41°34'47"N, 33°12'20"E	*C*. *coniocraea*	FUM, PRO	2
Turkey, Rize-İkizdere	40°38'18"N, 40°31'59"E	*C*. *furcata*	FUM, PRO	3
Turkey, Ordu, Çambaşı district, Road of Çambaşı plateau	40°44’06"N, 37°56’19"E	*C*. *furcata*	FUM, PRO	1
Portugal, Minho, Peneda	41°58'30" N, 08°13'39"W	*C*. *ramulosa*	FUM, PRO	5
Portugal, Minho, Castro Laboreiro	42°02'25"N, 08°09'50"W	*C*. *ramulosa*	FUM, PRO	2
Portugal, Beira Baixa, Proença-a-Nova	39°43'13"N, 7°51'23"W	*C*. *uncialis* subsp. *uncialis*	USN, SQUAM	1
Greece, Macedonia-Tracia, Polygyros	40°27’27”N, 23°19’18”E	*C*. *furcata*	FUM, PRO	2
Greece, Macedonia-Tracia, Agios Nikolaus	40°10’45”N, 23°49’10”E	*C*. *cercivornis*	FUM, PRO	2
		*C*. *furcata*	FUM, PRO	4
Greece, East Macedonia and Thrace, Thassos island	40°42'N, 24°39'E	*C*. *uncialis* subsp. *biuncialis*	USN, SQUAM	1
Croatia, Lika-senj, Mt. Velebit	44°30'02''N, 15°17'32''E	*C*. *furcata*	FUM, PRO	1
Russia, Caucasus, Krasnodar Territory, Mt. Fisht (LE)	43°57'46''N, 39°55'36''E	*C*. *pyxidata*	FUM, PRO	2
Russia, Caucasus, Krasnodar Territory, Mt. Armovka (LE)	43°52'28''N, 40°39'20''E	*C*. *coniocraea*	FUM, PRO	1
Russia, Caucasus, Karachaevo-Cherkesiya Republic, Teberda (LE)	48°28'10"N, 41°42'46"E	*C*. *pyxidata*	FUM, PRO	1
Sweden, Öland, Böda	57°15'N, 17°01'E	*C*. *rangiformis*	ATR, RANG, NRANG	1
Finland, Uusimaa, Espoo, Luukkaa Recreation Area	60°19'N, 24°39'E	*C*. *furcata*	FUM, PRO	2
		*C*. *phyllophora*	FUM, PRO	1
Finland, Southwest Finland, Salo	60°25'12"N, 23°9'12"E	*C*. *gracilis* subsp. *gracilis*	FUM, PRO	6
		*C*. *mitis*	FUM, PRO, USN	2
Finland, Uusimaa, Kirkkonummi	60°06'31"N, 24°26'38"E	*C*. *gracilis* subsp. *gracilis*	FUM, PRO	3
Finland, Uusimaa, Espoo, Ramsö	60°06'34"N, 24°42'30"E	*C*. *gracilis* subsp. *gracilis*	FUM, PRO	2
Finland, Tavastia Proper, Torronsuo	60°44'N, 23°43'E	*C*. *stygia*	ATR, FUM, PRO	1
Finland, Kainuu, Sotkamo (H)	64°07'45"N 28°23'28"E	*C*. *coniocraea*	FUM, PRO	1
Denmark, Faroe Island, Viðoy Island, Viðareiðy (H)	62°19'38''N, 6°29'36''W	*C*. *gracilis* subsp. *gracilis*	FUM, PRO	1
Denmark, Sjælland, Asserbo Plantage (H)	56°1'N, 11°59'E	*C*. *furcata*	FUM, PRO	1
		*C*. *rangiformis*	ATR, RANG, NRANG, FUM, PRO,	1
Portugal, The Azores, Flores, Reserva Florestal Natural das Caldeiras Funda e Rasa	39°24'05"N, 31°13'24"W	*C*. *rangiformis*	ATR, RANG, NRANG	3
Portugal, The Azores, Flores, Ponta Delgada	39°29'16"N, 31°11'00"W	*C*. *stereoclada*	FUM, PRO, BOU	2
Portugal, The Azores, Flores, Reserva Florestal Natural do Morro Alto e Pico da Se	39°26'17"N, 31°13'21"W	*C*. *rangiformis*	ATR, RANG, NRANG, FUM, PRO	3
Portugal, The Azores, Pico, Road EN3	38°29'50"N, 28°25'28"W	*C*. *stereoclada*	FUM, PRO, BOU	3
Portugal, The Azores, Pico Currais do Morais	38°28'41"N, 28°26'07"W	*C*. *stereoclada*	FUM, PRO, BOU	2
		*C*. *rangiformis*	ATR, RANG, NRANG, FUM, PRO	1
Portugal, The Azores, Pico, Cabeço Gordo	38°29'15"N, 28°27'18"W	*C*. *stereoclada*	FUM, PRO, BOU	1
		*C*. *rangiformis*	ATR, RANG, NRANG, FUM, PRO	2
Portugal, The Azores, Pico, EN3-EN2	38°28'50"N, 28°18'47"W	*C*. *stereoclada*	FUM, PRO, BOU	2
Portugal, The Azores, Pico, lakes	38°27'49"N, 28°17'05"W	*C*. *stereoclada*	FUM, PRO, BOU	2
Portugal, The Azores, Terceira, Road EN5-2A	38°42'48"N, 27°11'12"W	*C*. *rangiformis*	ATR, RANG, NRANG, FUM, PRO	2
Portugal, The Azores, Terceira, Fontinhas	38°43'41"N, 27°09'53"W	*C*. *rangiformis*	ATR, RANG, NRANG, FUM, PRO	2
			ATR, RANG, NRANG	1
Portugal, The Azores, Pico, Cais do Mourato	38°33'32"N, 28°28'20"W	*C*. *squamosa*	THAM, BAR	1
Portugal, The Azores, Pico, Baia das Canas	38°27'42"N, 28°13'58"W	*C*. *squamosa*	THAM, BAR	1
Portugal, Madeira island, Levada do Furado	32°44'12"N, 16°53'17”W	*C*. *stereoclada*	FUM, PRO, BOU	1
Canada, Yukon Territory, Alaska Hwy (H)	61°10'48"N, 135°22'52"W	*C*. *cornuta*	FUM, PRO	1
Canada, Yukon Territory, Klondike Hwy (H)	60°48’ 13"N, 137°26’03"W	*C*. *macroceras*	FUM, PRO	1
USA, Alaska, Unimak Island, 1.5 Km Airstrip (H)	54°50'33"N, 163°24'16"W	*C*. *crispata var*. *cetrariformis*	SQUAM	1
USA, Alaska, Unimak Island, 3 Km Airstrip (H)	54°50'13"N, 163°25'01"W	*C*. *crispata var*. *cetrariformis*	SQUAM	1
USA, Alaska, Unalga Island (H)	53°57'35"N, 166°11'11"W	*C*. *uncialis*	USN, SQUAM	1
USA, Alaska, Noatak Preserve (H)	68°28'N, 161°28'W	*C*. *gracilis* subsp. *vulnerata*	FUM, PRO	1
USA, Tennessee, Cocke Co. (H)	35°44’12"N, 83°14’29"W	*C*. *furcata*	FUM, PRO	1
Costa Rica, Cartago (H)	09°52'N, 83°55'W	*C*. *granulosa*	THAM	1
Russia, Primorye Territory, Zabolochennaya River (LE)	45°13'43''N, 136°31'05''E	*C*. *macilenta*	THAM	1
Russia, Primorye Territory, Golubichnaya River (LE)	44°54'20''N, 136°31'58''E	*C*. *cercicornis*	FUM, PRO	1

ATR = atranorin, BAR = barbatic acid, BOU = bourgeanic acid, FUM = fumarprotocetraric acid, NRANG = nor-rangiformic acid, PRO = protocetraric acid, RANG = rangiformic acid, SQUAM = squamatic acid, THAM = thamnolic acid, USN = usnic acid

Three loci were selected for the population–based study of *H*. *bachmannii*: ITS rDNA, LSU rDNA, and mtSSU. Of these, ITS rDNA and LSU rDNA were selected according to the results of Millanes et al. [6], who recommend the use of these loci as barcodes in Tremellomycetes. The following loci were tested to determine which of them was the most informative at population level: *l41* (with the primers L41F/L41R), IGS rDNA (with LR12R/5SRNA), *atp6* (with ATP6-1/ATP6-2), *rpb2* (with bRPB2-6F/bRPB2-11R), *rpb1* (with RPB1-Af/RPB1-CR), *ef1α* (with 983F/1567). So far no sequences of *H*. *bachmannii* were obtained for any of these loci. Either the amplification failed or the obtained sequences corresponded to the lichen mycobiont. Therefore we decided to use mtSSU, the only additional locus that worked.

In addition, DNA was extracted from one of the host species, *Cladonia rangiformis* Hoffm., trying to amplify the same loci used for *H*. *bachmannii*, such as recomended by Vienne et al. [21]. However, mtSSU was uninformative for *C*. *rangiformis*, whereby we amplified IGS rDNA, a highly variable locus that we had previously tested in several species of the genus *Cladonia* [22].

### DNA extraction, PCRs and sequencing

We cannot discard the coinfection of a single thallus of *Cladonia* by several strains of *H*. *bachmannii*. For this reason, we selected a single gall of each sample of *H*. *bachmannii* for the DNA extraction. The total DNA was extracted using E.Z.N.A. forensic DNA kit (Omega Bio-tek, Georgia, U.S.A.), according to the manufacturer's instructions. The DNA was eluted in the final step in 100 μl of elution buffer included in the kit. PCRs were carried out with Ready-to-Go-PCR Beads (GEHealthcare Life Sciences, UK). The volume of the reaction was 25 *μ*l with 3 *μ*l of extracted DNA. The primers used were: ITS1F/BasLSU3-3 [23–24] for ITS rDNA, BasLSU3-5/LR5 [24–25] for LSU rDNA, and MS1/MS2 [26] for mtSSU (primer sequences in S4 Table). The PCR programs for ITS rDNA and LSU rDNA were the same used by Millanes et al. [24], and for mtSSU: 95°C for 5 min; 35 cycles of 95°C for 30 s, 50°C for 30 s and 72°C for 1 min; with a final extension at 72°C for 10 min. The primers used to amplify *C*. *rangiformis* were: ITS1F/ITS4 [23, 26] for ITS rDNA, LROR/LR5 [25] for LSU rDNA, and IGSf/IGSr [27] for IGS rDNA. The PCR programs for ITS rDNA and IGS rDNA were described in Pino-Bodas et al. [28], and for LSU rDNA 95°C for 5 min; 30 cycles of 95°C for 30 s, 55°C for 30 s and 72°C for 1 min; with a final extension at 72°C for 10 min. PCR products were purified with E.Z.N.A. Ultra-Sep Gel Extraction Kit or ExoSAP-IT (USB Corporation, OH, USA). The sequencing was performed at Macrogen Europe service (www.macrogen.com), with the same primers as used for the PCR.

### Secondary metabolites of the hosts

All the host specimens were studied by thin layer chromatography (TLC) according to the standardized procedures [29–30], with the solvent systems A and B. The secondary metabolites of *C*. *rangiformis* were additionally studied by high–performance liquid chromatography (HPLC) and ultra–performace liquid chromatography–mass spectrometry (UPLC–MS) according to [9, 31–32]. The fatty acids of *C*. *rangiformis* were studied by gas chromatography–mass spectrometry (GC–MS). Secondary metabolites extractions, HPLC, GC–MS and UPLC–MS protocols are included as Supporting Information (S1 File).

### Phylogenetic analyses and haplotypes networks

The sequences were assembled in Sequencher 4.1.4 (GeneCodes, Ann Arbor, MI). The alignments were implemented in MAFFT [33] and BIOEDIT [34]. BLAST searches were done to verify the identity of the sequences. A few sequences corresponded to other *Tremellomycetes* genera and were deleted.

A phylogenetic analysis based on ITS rDNA and LSU rDNA was carried out to test whether *H*. *bachmannii* is monophyletic. To this end the following species were selected: two species of *Hetereocephalacria*, two species of *Syzygospora*, one species of *Piskurozyma*, three species of *Filobasidium*, three of *Goffeauzyma* (voucher specimens in Supporting Information, S1 Table). As outgroup *Cystofilobasidium bisporidii*, *C*. *capitatum* and *C*. *ferigula* were selected according to the results of Millanes et al. [26] and Weiss et al. [35]. The ambiguous regions were removed using Gblock [36] with the less stringent options. Each region and the combined dataset were analyzed by maximum likelihood (ML). The ML analyses were implemented using RAxML 7.0.3 [37] assuming the GTRGAMMA model. The node support was estimated with rapid bootstrap algorithm, using 1000 pseudoreplicates. Congruence between the loci was tested following Lutzoni et al. [38], manually checking the clades with at least 70% bootstrap support. No incongruity was detected.

Haplotype networks for each locus under statistical parsimony were constructed in TCS 1.21 [39], considering the gaps as a 5th character.

### Genetic structure analyses

Summary statisticals including haplotype diversity and nucleotide diversity were calculated in DnaSP v.5 [40]. In order to assess the contribution of the different potential factors (host species, geographical origin and host secondary metabolites) to the overall genetic variation of *H*. *bachmannii*, analyses of molecular variance (AMOVA) were conducted in ARLEQUIN V3.5 [41]. In these analyses only the groups containing more than five specimens were considered. To examine the relative contribution of these three factors, redundancy analyses (RDA) and partial redundancy analyses (pRDA) were run in R (R Development core Team 2017), using the *Vegan* package [42]. For these analyses a binary matrix with the haplotypes of *H*. *bachmannii* was used as dependent matrix. Other three binary matrices containing data of host species, host secondary metabolites (classified in chemotypes), and the geographical origin (The Azores, Southern Europe, Southern Finland, America, Asia) were used as explanatory matrices. The variation explained by each variable group was estimated using adjusted R2 because the number of variables in each matrix was not the same. The statistical significance was assessed using a permutation–based ANOVA test with 2000 permutations.

A bayesian clustering algorithm to estimate genetically homogeneous groups was implemented in STRUCTURE 2.3.4 [43]. Although this method was implemented to infer the population structure using unlinked markers, several studies have proved that the SNPS from sequences data as independent loci are also suited [44–45]. The analyses were run assuming an admixture model, without consideration of locality or host species of the specimens and allele frequencies independently modelled. The analysis was performed with five runs per K value (number of clusters), the range of K was from 1 to 20 with 100.000 iterations discarded as burnin and 2000.000 iterations kept for each replicate. The optimum value of K was calculated with the Evanno method in STRUCTURE HARVESTER [46–47]. The results of different runs were combined using CLUMPP 1.1.2 [48] and the barplots were generated with DISTRUCT 1.1 [49]. To assess whether the specimens of *H*. *bachmannii* on different species hosts, on different chemotypes or from different geographical regions were randomly distributed across the clusters, Chi-square tests were performed (http://www.physics.csbsju.edu/stats/). Only the groups (host species, secondary metabolites and geographical regions) with a frequency ≥ 5 were included.

### Host-parasite comparisons

To compare the genetic structure of the parasite and the host, we selected *Cladonia rangiformis* as host species for the following reasons: 1) the largest number of specimens of *H*. *bachmannii* was found on this host; 2) it has two different chemotypes, one with atranorin, rangiformic, and nor-rangiformic acids; another that, in addition to the above compounds, contains fumarprotocetraric and protocetraric acids, 3) the largest number of haplotypes of *H*. *bachmannii* found growing on this host.

The genetic variation for host and parasite was calculated in DnaSP v.5.

Three approaches were used in order to compare the genetic variation of *H*. *bachmannii* and *C*. *rangiformis*. Firstly, we performed partial Mantel test between pairwise [Fst/(1-Fst)] matrices of *H*. *bachmannii* and *C*. *rangiformis* correcting with geographic distances with 2000 random permutations using the VEGAN package in R. The pairwise geographical distance matrix was calculated with euclidean distances from the geographic coordinates using dist() function. Three different comparisons were tested: ITS–*H*. *bachmannii*/ITS–*C*. *rangiformis*, LSU–*H*. *bachmannii*/LSU–*C*. *rangiformis* and the combined dataset–*H*. *bachmannii*/combined dataset–*C*. *rangiformis*. Secondly, an analysis was run in STRUCTURE 2.3.4 with the same conditions specified above for the specimens of *H*. *bachmannii* and *C*. *rangiformis*. The congruence between the clusters of host and parasite defined by STRUCTURE was assessed by computing the proportion of individuals assigned together in the same cluster in both analyses. Contingency table analyses (http://www.physics.csbsju.edu/stats/) were used to test the association between the host chemotype and clusters generated by STRUCTURE. Variance analyses (ANOVA) in STATGRAPHICS 5.1 were carried out to study whether the clusters of *H*. *bachmannii* differed in the amount of phenolic compounds or fatty acids. Several ANOVAs were carried out, considering the total amount of substances (total amount of phenolic compounds, and total amount of fatty acids), the total amount of different subsets of fatty acids (saturated fatty acids, unsaturated acids and free fatty acids) and the amount of every phenolic substance separately (atranorin, fumarprotocetraric acid and protocetraric acid) and the amount of most abundant fatty acids separately (linoleic, oleic, palmitic and stearic acids). Bartlett test was used to check the variance homogeneity while Kolmogorov test checked the normality of the variables. All the variables had a homogeneous variance. Three variables were not normal (phenolic compounds total contents, fumarprotocetraric acid contents and protocetraric acid contents) and were analyzed by Kruskal-Wallis.

Finally, simple AMOVA analyses were made to study the genetic differentiation of *H*. *bachmanni*i and *C*. *rangiformis* among geographic regions and host chemotypes.

## Results

The 123 specimens of *H*. *bachmannii* were found on 20 host species (Table 1) with ten different chemotypes (Table 1). The chemotype containing fumarprotocetraric and protocetraric acids was the most abundant, found on 47 host specimens from ten species. HPLC and UPLC–MS analyses revealed 14 compounds for *C*. *rangiformis* (S1 File). The total contents of phenolic compounds varied from 19.1 to 61.5 mg/g of dry weight (d.w.). Atranorin was the major compound in all the populations except in one of them, where the contents of fumarprotocetraric acid exceeded that of atranorin. By means of GC–MS a total of 16 fatty acids methyl esters (FAME) derived from triglycerides and additionally two free fatty acids (FFA) were identified from *C*. *rangiformis* (S1 File).

In total 304, new sequences of *H*. *bachmannii* and 90 of *C*. *rangiformis* were generated (S5 Table). In the phylogenetic analysis, all the specimens of *H*. *bachmanii* formed one strongly supported clade (S1 Fig), into which another lichenicolous species, *H*. *physciacearum* was grouped. The haplotype and nucleotide diversity values are shown in Table 2.

**Table 2:** Statistical summary of genetic variation of *Heterocephalacria bachmannii*.

	N	H	Hd	π
**ITS rDNA**	112	28	0.877	0.00559
America	8	7	0.964	0.00679
The Azores	29	5	0.643	0.00431
Southern Europe	54	15	0.901	0.00713
Southern Finland	20	6	0.579	0.00342
**LSU rDNA**	121	24	0.913	0.00251
America	8	5	0.893	0.00491
The Azores	29	3	0.599	0.00176
Southern Europe	60	13	0.905	0.00306
Southern Finland	21	8	0.767	0.00145
**mtSSU**	72	6	0.611	0.00393
America	3	1	0.000	0.00000
The Azores	14	4	0.659	0.00377
Southern Europe	42	3	0.516	0.00347
Southern Finland	13	1	0.000	0.00000

N = number of sequences, H = number of haplotypes, Hd = haplotype diversity, π = nucleotide diversity.

**Fig 1:**
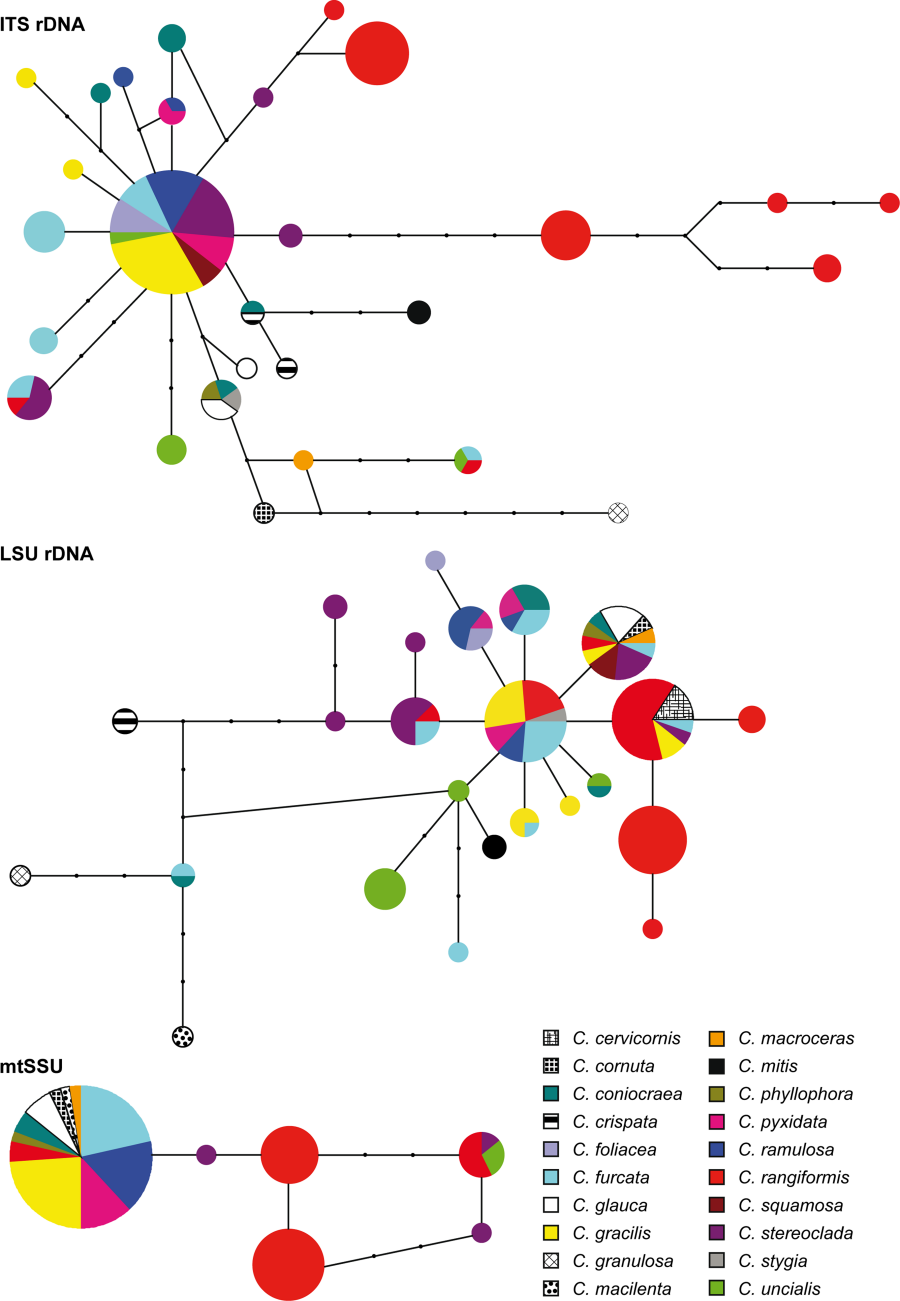
Haplotype networks of *Heterocephalacria bachmannii* inferred by TCS for the three loci. Each circle represents a haplotype, the circle size is proportional to haplotype frecuency. Small circles represent haplotypes not observed in the data. The colors represent the different host species.

For each of the three loci, the haplotypes were connected in a unique network with 95% of confidence (Fig 1). A total of 28 ITS rDNA haplotypes, 24 of LSU, and six of mtSSU were found. The distribution of haplotypes among host species revealed that six ITS rDNA haplotypes, nine LSU rDNA haplotypes, and two mtSSU haplotypes were present on multiple host species, while 22 ITS rDNA haplotypes, 19 LSU rDNA haplotypes and four mtSSU haplotypes restricted themselves to a unique host species. Five haplotypes of ITS rDNA, seven of LSU rDNA, and two of mtSSU were present in several geographical regions. Southern Europe showed the highest number of haplotypes: 19 in ITS rDNA, 14 in LSU rDNA, and three in mtSSU (Table 2). In the ITS rDNA haplotype network, the most abundant haplotype represented 32.1% of all the specimens, and it was found on several host species (Fig 1). A group of haplotypes restricted to *C*. *rangiformis* was separated by four mutational steps (Fig 1A). Another haplotype found on *C*. *granulosa* also separated from the rest by five mutational steps. In LSU rDNA network, the most abundant three haplotypes represented 44% of all the specimens, and they were found on several host species (Fig 1B). One haplotype restricted only to *C*. *granulosa* and another one only to *C*. *macilenta* were the most distant. In mtSSU haplotype network, the most abundant haplotype represented 58% of individuals, and it was present on 11 host species (Fig 1C).

### Genetic structure of *Heterocephalacria bachmannii*

The results of AMOVA analyses are shown in Table 3. Most of the varition was found among the chemotypes (33.3–73.1%) and among host species (29.0–68.5%) while the genetic differentiation among geographical regions explained less percentage of variation (7.0–40.3%).

**Table 3:** Analyses of molecular variance (AMOVA) with the host species, chemotype of the host and geographical region as grouping factors for each loci.

	d.f.	SS	Variance components	% of variation	Fst
**ITS rDNA**					
Among host species	7	62.40	0.654	29.017	0.290***
Within host species	87	140.63	1.601	70.982	
Among chemotypes	6	77.5	0.8511	35.779	0.358***
Within chemotypes	101	153.57	1.52769	64.221	
Among regions	3	18.272	0.151	6.965	0.069***
Within regions	113	232.79	2.029	93.034	
**LSU rDNA**					
Among host species	7	41.30	0.468	33.132	0.331***
Within host species	84	61.57	0.946	66.867	
Among chemotypes	6	45.49	0.513	33.278	0.332***
Within chemotypes	98	83.73	1.030	66.721	
Among regions	3	15.250	0.169	11.037	0.110***
Within regions	106	125.90	1.362	88.962	
**mtSSU**					
Among host species	5	45.73	0.803	68.506	0.685***
Within host species	53	19.57	0.369	31.493	
Among chemotypes	3	38.10	0.836	73.120	0.731***
Within chemotypes	64	19.68	0.307	26.879	
Among regions	2	19.72	0.479	40.252	0.402***
Within regions	66	47.00	0.712	59.747	

d.f. = degrees of freedom, SS = sum of squares.

*** significant results with *P–values* < 0.001.

**Fig 2:**
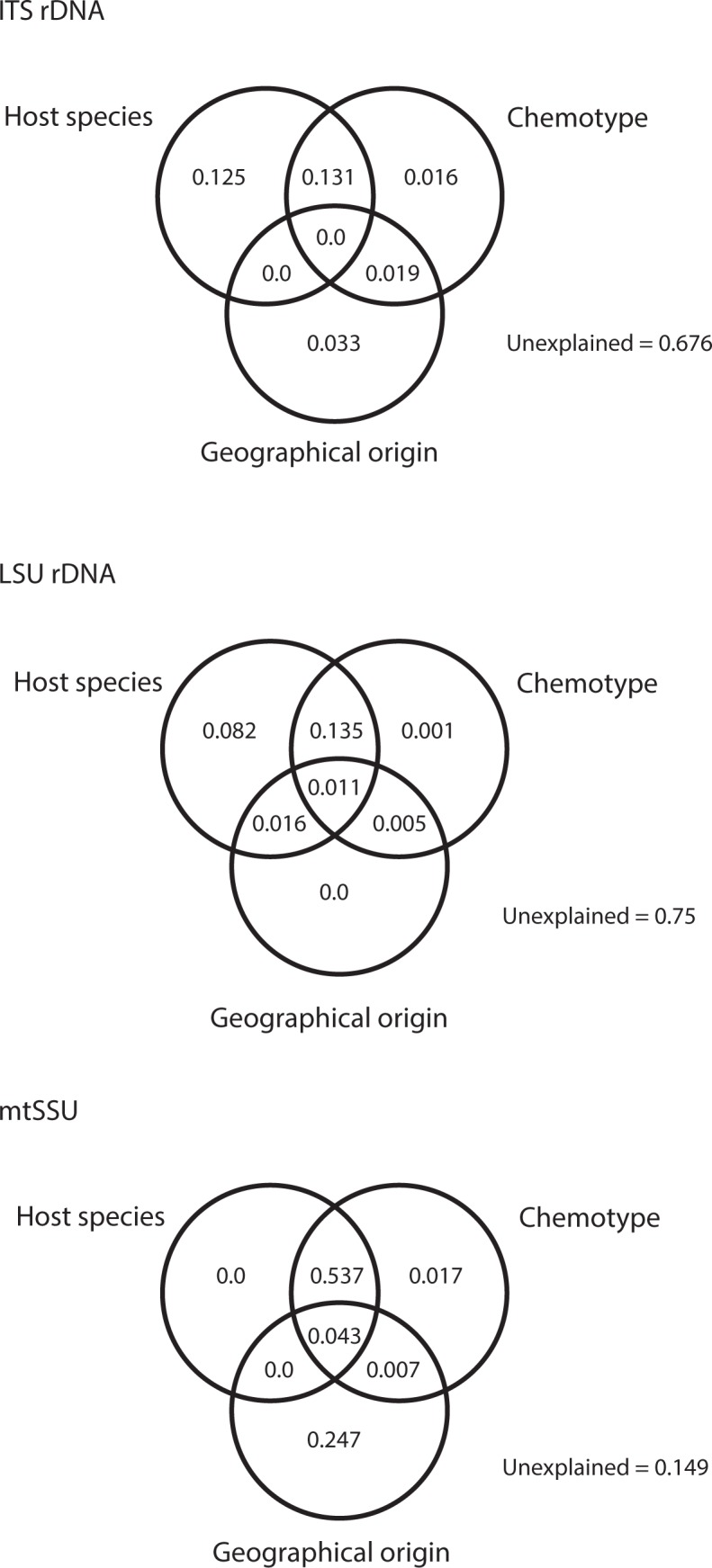
Diagrams show the results of redundancy analyses of *Heterocephalacria bachmannii*. Fractions of genetic variance explained by host species, chemotype of the host and geographical origin.

Fig 2 shows the results of the redundancy analyses. The pRDAs show that the proportion of the genetic variation explained by the chemotypes, the host species or the geographical origin, when the other factors are kept under control, was very small. The greatest proportion of genetic variation was explained by the host species in conjunction with the chemotype (0.13 in ITS rDNA, 0.13 in LSU rDNA and 0.53 in mtSSU). The geographic origin on its own explained only a little genetic variation in ITS rDNA and LSU rDNA, while in mtSSU it explained a greater proportion than each of the other two factors. All the three variables, jointly taken, explained only a small proportion of the genetic variation in the three loci.

**Fig 3:**
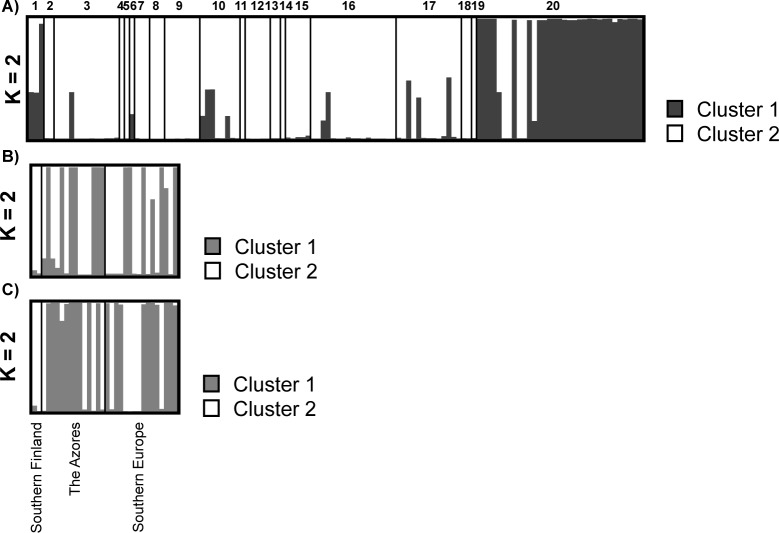
Population clusters obtained from multilocus analyses using STRUCTURE. The vertical bars represent the individuals, the colors indicate the proportion of the genome assignable to each cluster. Populations are separated by vertical black lines. (A) Resulting clusters of *H*. *bachmannii* on all host species. The numbers represent the different hosts: 1. *C*. *cervicornis*, 2. *C*. *crispata*, 3. *C*. *gracilis*, 4. *C*. *cornuta*, 5. *C*. *macroceras*, 6. *C*. *granulosa*, 7. *C*. *foliacea*, 8. *C*. *glauca*, 9. *C*. *ramulosa*, 10. C. *uncialis*, 11. *C*. *phyllophora*, 12. *C*. *pyxidata*, 13. *C*. *mitis*, 14. *C*. *stygia*, 15. *C*. *coniocraea*, 16. *C*. *furcata*, 17. *C*. *stereoclada*, 18. *C*. *squamosa*, 19. *C*. *macilenta* and 20. *C*. *rangiformis*; (B) Resulting clusters of *H*. *bachmannii* collected on *C*. *rangiformis;* (C) Resulting clusters of *C*. *rangiformis* parasitized by *H*. *bachmannii*.

The clustering algorithm implemented in STRUCTURE revealed that the optimal number of clusters was two (highest values of ΔK were obtained for K = 2). The 90.9% of individuals were assigned to one cluster with membership coefficients > 0.7. Ten speciemens could not be assigned to any of the clusters (membership coefficients < 0.7). The inviduals were nonrandomly distributed in the two genetic clusters (S2 Table). Different association with respect to the host species (Chi–square = 68.4, d.f. = 7, *P*–*value* ≤ 0.001), chemotypes (Chi–square = 78.5, d.f. = 6, *P*–*value* ≤ 0.001) and the geographical region (Chi–square = 14.4, d.f. = 4, *P*–*value* ≤ 0.01) was found. One cluster contained specimens on *Cladonia rangiformis* (n = 26), *C*. *furcata* (n = 1) and *C*. *cervicornis* (n = 1). All the specimens of this cluster were collected in Southern Europe and in the Azores. The other cluster was associated with the others host species, although five specimes were also associated with *C*. *rangiformis* as the host species (Fig 3). The specimens assembled in this cluster were collected in all geographic regions and chemotypes.

### Comparison of *Heterocephalacria bachmannii* with *Cladonia rangiformis*

Table 4 shows the genetic variation of *H*. *bachmannii* (collected on *C*. *rangiformis*), as well as that of the host species itself. The results of partial Mantel test are shown in Table 5. *Cladonia rangiformis* and *H*. *bachmannii* pairwaise Fst values were not significantly correlated.

**Table 4:** Statistical summary of genetic variation of *H*. *bachmannii* on *C*. *rangiformis* and *Cladonia rangiformis*.

	N	H	Hd	π
***H*. *bachmannii***				
**ITS rDNA**	31	8	0.770 (0.00233)	0.00734
The Azores	14	4	0.676 (0.00484)	0.00605
Southern Europe	15	5	0.638 (0.00867)	0.00583
**LSU rDNA**	32	7	0.664 (0.00239)	0.00099
The Azores	14	5	0.725 (0.01082)	0.00137
Southern Europe	16	5	0.513 (0.00675)	0.00059
**mtSSU**	23	4	0.676 (0.00389)	0.00328
The Azores	10	2	0.509 (0.01015)	0.00314
Southern Europe	12	2	0.167 (0.01804)	0.00068
***C*. *rangiformis***				
**ITS rDNA**	31	6	0.579 (0.00903)	0.00870
The Azores	13	4	0.731 (0.00773)	0.01092
Southern Europe	17	5	0.647 (0.01405)	0.00914
**LSU rDNA**	28	11	0.812 (0.00415)	0.00244
The Azores	14	7	0.782 (0.01093)	0.00146
Southern Europe	14	8	0.901 (0.00331)	0.00539
**IGS rDNA**	31	10	0.569 (0.01122)	0.00490
The Azores	15	4	0.275 (0.0220)	0.00298
Southern Europe	15	7	0.781 (0.01031)	0.00626

N = number of specimens, H = number of haplotypes, Hd = haplotype diversity and variance in brackets, π = nucleotide diversity.

**Table 5:** Results of partial Mantel tests between *H*. *bachmannii* and *Cladonia rangiformis*.

Compared dataset	r	*P-value*
ITS rDNA	0.1973	0.15642
LSU rDNA	-0.1063	0.47726
Combined datasets	0.1023	0.28086

*P-value* based on 2000 permutations.

The STRUCTURE analysis carried out with *H*. *bachmannii* samples collected on *C*. *rangiformis* showed a ΔK peak at a K = 2, as did the STRUCTURE analysis of *C*. *rangiformis* (Fig 3). In both analyses all the specimens were assigned to one of the two clusters with values > 0.8, but the cluster composition was different. The clusters defined by STRUCTURE for *H*. *bachmannii* and *C*. *rangiformis* were less congruents, only 43.8% of specimens were assigned to the same cluster. The contingency table analyses indicated that there is not a significant association between the host chemotypes and the *H*. *bachmannii* clusters (S3 Table) generated in STRUCTURE (Chi–square = 0.406, d.f. = 1, *P–value* = 0.524). ANOVA and Kruskal–Wallis analyses did not find significant differences in the contents of the host secondary metabolites between the two clusters of *H*. *bachmannii* (Table 6).

**Table 6:** Results of ANOVA analyses searching for associations of STRUCTURE clusters of *H*. *bachmannii* and the concentration of secondary metabolites of the host.

	F/Statistic	*P-value*
**Phenolic substances**		
Atranorin	4.00	0.0709
Fumarprotocetraric acid^a^	0.81	0.3662
Protocetraric acid^a^	1.45	0.2283
Total content of phenolic substances^a^	2.59	0.1073
**Fatty acids**		
Total content of fatty acids	0.91	0.3622
Total content of saturated fatty acids	0.08	0.7816
Total content of unsaturated fatty acids	1.12	0.3157
Total content of free fatty acids	0.39	0.5465
Linoleic acid	0.48	0.5025
Oleic acid	0.82	0.3873
Palmitic acid	0.12	0.7352
Stearic acid	0.66	0.4356

^a^ Variable analyzed by Kruskal-Wallis

The AMOVA analyses are shown in the Table 7. Significant genetic differenciation was found between *H*. *bachmannii* on different host chemotypes and geographical regions. However, the variance explained by the geographical origin (31.7% in ITS rDNA, 15.9% in LSU rDNA and 58.40% in mtSSU) was higher than the explained by the chemotypes (16.8% in ITS rDNA, 13.6 in LSU rDNA and 15.7% in mtSSU). The genetic variance of *C*. *rangiformis* from different geographical origins was only significantly different in LSU rDNA analysis, while no significant differences were found in any loci between specimens with different chemotypes.

**Table 7:** Analyses of molecular variance (AMOVA) of *H*. *bachmannii* on *C*. *rangiformis* host species and *C*. *rangiformis*.

	d.f.	SS	Variance components	% of variation	Fst
***H*. *bachmannii***					
**ITS rDNA**					
Among chemotypes	1	9.607	0.48300	16.78928	0.16789 ***
Within chemotypes	28	67.027	2.39381	83.21072	
Among regions	1	2.800	0.16317	31.65025	0.31650 ***
Within regions	28	9.867	0.35238	68.34975	
**LSU rDNA**					
Among chemotypes	1	2.287	0.13454	13.62065	0.13621 **
Within chemotypes	28	18.657	0.85321	86.37935	
Among regions	1	2.319	0.14680	15.89882	0.15899 **
Within regions	27	15.714	0.77657	84.10118	
**mtSSU**					
Among chemotypes	1	2.285	0.13567	15.71543	0.15715 *
Within chemotypes	21	15.280	0.72763	84.28457	
Among regions	1	7.773	0.66364	58.40000	0.58400 ***
Within regions	20	9.455	0.47273	41.60000	
***C*. *rangiformis***					
**ITS rDNA**					
Among chemotypes	1	0.806	-0.17895	-5.56072	-0.05561 ns
Within chemotypes	28	91.281	3.39706	105.56072	
Among regions	1	3.582	-0.09555	-1.95272	-0.01953 ns
Within regions	28	135.938	4.98877	101.95272	
**LSU rDNA**					
Among chemotypes	1	0.985	-0.06634	-3.87251	-0.03873 ns
Within chemotypes	25	41.329	1.77935	103.87251	
Among regions	1	5.382	0.22208	7.83240	0.07832 ***
Within regions	25	62.487	2.61336	92.16760	
**IGS rDNA**					
Among chemotypes	1	0.476	0.01787	7.51662	0.07517 ns
Within chemotypes	27	5.938	0.21991	92.48338	
Among regions	1	1.017	-0.00228	-0.21762	-0.00218 ns
Within regions	27	28.362	1.05044	100.21762	

*** significant results with *P–values* < 0.001

** significant results with *P–values* < 0.01

* significant results with *P–values* < 0.05, ns no significant results with *P–values* > 0.05.

## Discussion

Owing to the high number of host specific lichenicolous fungi, it has been assumed that these fungi and their lichen hosts have coevolved [2]. Coevolution depends on the genetic variation and on the genetic structure of interacting species [50]. In a coevolution process, the parasite populations would be expected to have a structure determined by the hosts, and with a low gene flow among the host species [51]. Thus far, however, the investigations on the relations between lichenicolous fungi and lichens are still too poor. The genetic variation of *H*. *bachmannii* has been studied here for the first time in a broad geographic range. The data indicate that the genetic differentiation of *H*. *bachmannii* is related to the host secondary metabolites and the host species, while this species presents few geographic isolation. This study supports the presence of two genetic clusters of *H*. *bachmannii* specialized to different hosts.

### Genetic variation and genetic structure of *Heterocephalacria bachmannii*

The number of haplotypes in ITS rDNA and LSU were greater in southern Europe than in the other regions, an expected result considering that the sampling and the number of host species were higher in this region than in the other ones (Table 1). Our results also show mtSSU was less variable than the nuclear loci, as to the number of haplotypes, haplotype diversity, and nucleotide diversity. This result is concordant with prior studies [52–56] conducted in other fungi, the authors considered that the mutational rate in the mitocondrial genome is lower than in the nuclear genome. Nevertheless, this result could be also attributed to the fact that the number of mtSSU sequences obtained was smaller than the number of sequences of the other two loci. To check whether this explanation was plausible, we calculated the number of haplotypes for the nuclear loci taking into account only the specimens for which a sequence of mtSSU was obtained (data not shown). However, the number of haplotypes (20 in ITS rDNA and 15 in LSU rDNA) continued being smaller in mtSSU.

According to our results, the host species and the host secondary metabolites are the most relevant factors in the genetic structure of *H*. *bachmannii*, while the populations of *H*. *bachmannii* from different geographical regions show slight genetic differentiation. It supports the presence of gene flow among the populations separate by long distances. Similar results have been found for other lichenicolous fungi of the genus *Tremella* [5, 57]; while the opposite occurs in *Marchandiomyces corallinus* [4]. According to Werth et al. [5] the generalist lichenicolous fungi might be geographically structured, while the more specialist species might be structured by the host species. Therefore, *H*. *bachmannii* is expected to be structured by its hosts, all of which belong to the genus *Cladonia*. In other basidiomyceteous non-lichenicolous fungi, populations not geographically structured have been frecuently found [58–62]. It is assumed that fungal spores have an ability for long distance dispersal. However in some cases, no correlation has been found between the genetic and geographic distances [59] and other hypotheses have been proposed. Kretzen et al. [59] proposed two hypotheses to explain the absence of geographic structure in ectomycorrhizal fungi of the genus *Rhizopogon*. The first holds that competence can prevent offspring from establishing close to parents. The second hypothesis asserts that the mating systems could strongly support the outcrossing, whereby the spores coming from other populations would have a higher probability of survival success. Owing to our limited knowledge about the reproductive biology of *H*. *bachmanii*, we can not be sure that either hypothesis explains our results. Therefore, further studies are required to determine dispersal mechanisms of this species.

The effect of the lichen secondary metabolites on the lichenicolous fungi had been previously studied. Lawrey [63] found that certain phenolic compounds inhibit the growth of these fungi. Other lichenicolous fungi can only colonize the host species if another fungus has previously degraded those compounds [13]. It is also proved that the thalli of parasitized lichens have a lower concentration of phenolic substances than the non-parasitized ones [64]. However, ours is the first study in which genetic divergence among the populations of a lichenicolous fungus has been proved to be associated with the host secondary metabolites. Werth et al. [5] demonstrated that the host species was the factor that explained most of the data variance in *T*. *lobariacearum*, suggesting that the host species could create a selective environment that only certain strains would have the ability to infect. This particular environment might be created by the secondary metabolites of the host species [5]. Therefore it was to be highly expected that the genetic structure of the parasite should be strongly influenced by the secondary metabolites of the host. Even though the AMOVA results indicated that secondary metabolites explained slightly more genetic variation of *H*. *bachmannii* than the host species, in the redundance analyses the conjuntion of both factors explained most of the genetic variation. The lack of resolution in our analyses may be due to the low intraspecific chemical variation of the hosts infected by *H*. *bachmannii*. Although several chemotypes are known for many of the host species studied [22], only one parasitized host species had several chemotypes (*C*. *rangiformis*), what makes difficult to separate both effects, “host species” and “host secondary metabolites”. We do not know if our sampling was biased towards one chemotype, or if *H*. *bachmanii* in some *Cladonia* species only parasitizes one chemotype. Despite this parasite is not a rare species [17–18], there are no data about its distribution on the chemotypes within the host species and the total range of secondary metabolites it tolerates.

It is also remarkable that the redundacy analyses showed high unexplained genetic variation in *H*. *bachmannii*. Therefore, other envarionmental factors could also be important in the genetic structure of this lichenicolous fungus.

### Discordant genetic structure between *Heterocephalacria bachmannii* and *Cladonia rangiformis*

The results also point out that the genetic structures of the host and the parasite are different. The populations of *H*. *bachmannii* were genetically more variable in the Azores than in Southern Europe, while the populations of *C*. *rangiformis* were more variable in Southern Europe. In addition, the differentiation among populations from different geographical regions was higher for *H*. *bachmannii* than for *C*. *rangiformis* (Table 7), which reveals a lower gene flow among the populations of the parasite than among those of the host. The differences between the genetic structure of the parasite and their hosts are frequent [65–67], which could be due to differences in evolutionary rates, differences in dispersion rates or movement of *H*. *bachmannii* among the different potential hosts. Mantel test did not reveal any significant correlation between the genetic distances of the host and the parasite, which indicate the absence of codispersion. Therefore, the hypothesis that the galls of *H*. *bahchmannii* were dispersed together with thallus fragments of *C*. *rangiformis* is not supported by our data. The potential movement among different host species could explain why the geographical structure of *H*. *bachmannii* is more accentuated when only the specimens on *C*. *rangiformis* are considered. Other plausible explanation would be that the lineage of *H*. *bachmannii* (specialized on *C*. *rangiformis*) colonized once the Azores and subsequently has extended across the potential hosts in the islands.

The host chemotypes have less influence on the genetic structure of *H*. *bachmannii* on *C*. *rangiformis* (Table 7) than expected. These chemotypes differ in the presence or absence of fumarprotocetraric acid, a compound synthesized by a number of *Cladonia* species [19–20] that act as hosts for *H*. *bachmannii*. Most likely a large number of genotypes of *H*. *bachmannii* are tolerant to this substance. The genetic structure of the lichenicolous fungi is neither shaped by the amount of phenolic compounds nor by the concentration of fatty acids (Table 6), indicating that the two genetic clusters tolerate a wide range of these compounds.

No evidence of geographical isolation was found between the populations of the Southern Europe and those of the Azores in *C*. *rangiformis*. *Cladonia* species present different genetic structure patterns. While Printzen & Ekman [68] found that populations of *C*. *subcervicornis* separated by a few kilometers were genetically isolated, in other species the populations were geographically weakly structured [69–71]. The most extreme case was that of *C*. *arbuscula* and *C*. *mitis*, two species with a bipolar distribution, the specimens of which appear intermixed in the northern and the southern hemispheres [72]. It is assumed that the predominant reproduction in *C*. *rangiformis* is asexual by dispersion of thallus fragments, because the apothecia are rare. The propagule size can directly influence the population structure of the species. Since several studies show that thallus fragments are dispersed at close range by wind [73–74], one would expect that the species with this type of reproduction should have a marked geographic structure in its populations. However, this is not always the case; in species of *Lobaria* with a varying propagule size, no correlation has been found between the propagule size and the geographic structure [75]. In *C*. *rangiformis*, gene flow between the populations of the two geographic regions could have two explanations. First, long distance dispersion of thallus fragments by the wind or other vectors. In fact, some studies have proved that birds can act as dispersion vectors for lichen fragments [76–77]. Second, that although the sexual reproduction is less frequent, the ascospores (which easily disperse long distance by the wind) could have a higher establishment success than the thallus fragments. The success of the propagules in developing new thalli is highly depending on the substrate on which they fall, the substrate formed by bryophytes being the most suitable in *C*. *mitis* [78].

## Supporting information

S1 File Secondary metabolites of *Cladonia rangiformis*. The analytical protocols, results, tables and references are decribed. Phenolic compounds were identified using HPLC and UPLC-MS, and fatty acids using GC-MS.
(DOC)

S1 Fig Maximum likelihood tree estimated from the concatenated dataset of ITS rDNA and LSU rDNA. (PDF)

S1 Table Collection information for the specimens included in the phylogenetic analysis. (DOC)

S2 Table Assignment of *H*. *bachmannii* specimens to the clusters inferred in STRUCTURE. All the specimens had membership coefficients ≥ 0.7 and they were assigned to cluster 1 or 2 without uncertainty. Chemotypes, host species and geographical origin.
(DOC)

S3 Table Distribution of *H*. *bachmannii* specimens on *C*. *rangiformis* in the clusters generated by STRUCTURE: Chemotypes and geographical origin. (DOC)

S4 Table List of primers used in PCR and sequencing reactions. (DOCX)

S5 Table GenBank accession numbers of *Heterocephalacria bachmannii* and *Cladonia rangiformis*. (XLSX)
