# Supplementary material for: Genetic variation and factors affecting the genetic structure of the lichenicolous fungus Heterocephalacria bachmannii (Filobasidiales, Basidiomycota)
Source: PLoS One. 2017 Dec 18;12(12):e0189603. doi: 10.1371/journal.pone.0189603 (PMC5734755; doi:10.1371/journal.pone.0189603)
Supplement: S1 File — The analytical protocols, results, tables and references are decribed. Phenolic compounds were identified using HPLC and UPLC-MS, and fatty acids using GC-MS. (DOC) [file pone.0189603.s001.doc]

**S1 File. Secondary metabolites of *Cladonia rangiformis***

**Material & Methods**

Phenolic compounds for HPLC analyses were extracted from 20 mg of dry lichen thallus (after removing the parasite), homogenized by a mortar and soaked in 500 µl of acetone at 50 ºC during 30 min. The samples were then centrifuged and the solids removed. Acetone was evaporated under nitrogen flow and extract was immediately dissolved in 500 µl of methanol. The samples were kept at 4 ºC until HPLC analysis. The analyses were carried out using a Waters™ HPLC system (Waters Corporation, Milford, MA, USA), consisting of a Millenium^32^ chromatography manager, 717 autosampler, 2996 photodiode array (PDA) detector, and 600 controller and a pump connected to an on-line degasser. The HPLC separations were performed on a Spherisorb ODS2 column (4.6 mm × 100 mm, particle size 5 µm), using (A) 0.5% *o*-phosphoric acid (85%, E. Merck, Darmstadt, Germany) in Milli Q water (Merck Millipore), and (B) HPLC grade methanol as solvents. The injection volume was 20 µl. The gradient elution with a flow-rate of 0.8 ml/min increased solvent B as follows: 0-15 min, 30-70%; 15-35 min, 70-100%; 35-50 min, 100%; 50-55 min, 100-30%; 55-65 min, 30%.

The UV-vis spectra and absorption maxima of sample components were recorded at 200-550 nm and the peaks were detected at 254 nm. The substances were identified using the UV spectrum and the retention time. The concentration of the lichen substances was determined using an external standard. The stock solution was prepared using benzoic acid as standard in methanol, the final concentration was 10mg/10 ml. The stock solution was diduted several times to prepare the calibration curve. Calibration curves were constructed by plotting the peak areas versus concentrations of the standard. The equation for the calibration curve was y = 1.0809x+1.5166 and the correlation coefficient was 0.9996. The analyses were performed in duplicate, and the average amount of secondary metabolites was used for the data analyses.

From the extracts prepared in methanol as above for HPLC analysis three samples, showing different profiles, were taken for a qualitative UPLC-MS analysis. The runs were performed on a Waters Q-Tof Premier mass spectrometer combined with an Acquity Ultra Performance LC^TM^ (UPLC). The column (at 40 °C) was an Acquity UPLC^TM^ BEH C18 2.1 × 100 mm with 1.7 μm particles. The solvent system consisted of 0.1% acetic acid and (B) acetonitrile (UPLC-MS grade). The gradient started from 5% B, reached 90% B in 11 min and remained there for 2 min before reconditioning. The flow rate was 0.300 ml/min and the injected amount 5.0 μl (Acquity Sample Organizer, at 10 °C). Samples were run by using electrospray ionisation technique (ESI) in negative ion modes. The source and desolvation temperatures were 150 and 400 °C, respectively. The data (centroid) were collected at a mass range of m/z 80-700 with scan duration of 1 s. In the identifications, the mass spectra were compared with literature [1–2].

For fatty acid analyses by GC-MS, 10 mg powdered dry sample were spiked with internal standards (IS) TG(17:0/17:0/17:0) and C17:0 free fatty acid (FFA), and mixed with 1 ml petroleum ether (bp 40-60 °C). Transesterification was performed by adding 250 µl of 0.5 N NaOMe in MeOH and a couple of boiling stones, and the mixture was boiled at 45 °C for 5 min. The samples were acidified with 15% NaHSO_4_ (0.5 ml) and the methyl esters as well as free fatty acids were extracted twice with petroleum ether (2 × 0.5 ml). The separated petroleum ether layers were combined, evaporated under nitrogen flow and dissolved into 50 µl of petroleum ether.

Fatty acid methyl esters (FAMEs) were analyzed on a Hewlett-Packard (HP) 5890 GC combined with an HP 5970 mass selective detector. A polar Stabilwax capillary column (25 m x 0.25 mm x 0.25 μm; Restek, Bellefonte, PA, USA) was used with helium as the carrier gas (0.5 mL/min) and split ratio of 15:1. The temperatures of the injector and MS source were both 240 °C. The oven temperature raised from 150 °C (3.25 min) to 240 °C at a rate of 10 °C/min; total run time was 30 min. The samples (3 µl) were injected by using Agilent 6850 automatic liquid sampler and the data were collected in EI mode (70eV) at a mass range of m/z 40-400. Identification of fatty acids and other lichen compounds was based on Wiley GC-MS and Nist 08’ libraries and on literature [1–3].

**Results**

**Phenolic compounds**

Table 1 shows the 14 compounds found in *C. rangiformis* by means of HPLC and UPLC-MS. Only six of them could be identified, when comparing the retention time, the UV spectra or the fragment ions with those published by other authors [1–3]. Rangiformic acid, nor-rangiformic acid and 1-methyl 3,4-dicarboxy-hexadecanoate do not absorb UV [4] and therefore they could not be detected by HPLC. The total contents of phenolic compounds varied from 19.1 to 61.5 mg/g of dry weight (d.w.). Atranorin was the major compound in all the populations except in one of them, where the contents of fumarprotocetraric acid exceeded that of atranorin. The concentration of atranorin varied from 17.4 to 27.1 mg/g d. w. These results are in agreement with Huovinen et al. [5] and Huneck et al. [2]. The concentration of fumarprotocetraric and protocetraric acids varied from 0.9 to 29.7 and from 0.02 to 1.6 mg/g d. w. respectively. We found the same fragment ions as detected in UPLC–MS for the different compouds by Huneck et al. [2], but the relative proportions of the fragments were different, probably owing to the different collision energy used.

The variation in the contents of phenolic compounds among the different samples of *C. rangiformis* parasitized by *H. bachmannii* was high (1.9-6.1 mg/g d. w. = 0.2-0.6 % d. w.). By means of UPLC-MS, six compounds could be identified when comparing the retention time, the fragment ions and the molecular weights with those reported earlier in *C. rangiformis* [2]. Huneck et al. [2] found five unkown compounds which were also present in our analyses (Table 1). We have obtained the UV spectrum for these compounds (suplementary material), not provided by Huneck et al. [2], but we could not identify them either. We found the same UV spectra in HPLC and UPLC-MS for three components eluted in the same order in both methods, whereby we conclude they are the same compounds (Table 1).

The concentration of phenolics compounds substantially varies among specimens in several species of *Cladonia,* for example in *C. stellaris* it varies from 1 to 5% [6–9], and in *C. rangiferina* from 1.0 to 2.8% [6]. It is proved that UV radiation, [10–11], latitude [6], age of the thalli [6, 12], and physical changes caused by reindeers [8] alter the concentration of phenolic substances in *Cladonia*. In addition, the concentration of phenolic compounds could be related to the time elapsed since the last rain. Although most of the phenolic compounds synthesized by lichens are poorly soluble in water, rainwater washes a certain amount of them, and that gathers in soil (e.g. fumarprotocetraric and protocetraric acids) [12–14].

**Fatty acids contents**

In this work, the profile and the concentration of fatty acids in *C. rangiformis* have been studied for the first time. Table 2 shows the fatty acids composition obtained by GC-MS analysis. We compared the retention times and the mass spectra of the compounds found with those recorded in the fatty acid library of our laboratory and with the libraries previously mentioned. A total of 16 fatty acids bond to triglycerides (TG) were identified as fatty acid methil esters (FAME) and additionally two free fatty acids. Out of the FAMEs, nine were saturated, three monounsaturated, two diunsaturated and two polyunsaturated fatty acids (Table 2). The two FFAs found were one saturated and the other diunsaturated compound. The total contents of fatty acids varied from 1.5 to 6.0 μg/mg. The major fatty acid was linoleic acid (18:2n-6), with a concentration varying between 0.51 and 1.87 μg/mg. Among the saturated fatty acids, palmitic acid (16:0) was the most abundant, with a concentration varying between 0.12 and 0.48 μg/mg. The fatty acid profiles studied here are similar to those reported in other species of the genus [15–18]. However, there are a few studies that quantify the concentration of fatty acids in lichens, particularly in the genus *Cladonia*; but if we compare our values with the ones given by Vu et al. [18], we find that ours were much higher. The high content in our samples is likely caused by them being infected by *H. bachmannii*. In addition, linoleic acid was found in the highest concentration (Table 2), a fatty acid that increases its concentration in fungi whenever they are parasitized [19]. It has been observed in several plant species that they increase the production of fatty acids as a defence against parasitic fungi [20–22]. It could be possible for lichens to use a similar defence mechanism.

By means of GC–MS, three other compounds (not fatty acids) were also detected: atraric acid, phytol and o-Orcellinaldehyde, substances that absorb UV light but could not be detected by HPLC. This might be due to the very low content of this compounds and therefore they are only detectable by GC–MS, a technique more sensitive than HPLC. Another possibility is that atraric acid and phytol were produced during the esterification process. Atraric acid is a simple acid resulting from atranorin hydrolysis [23], while the phytol is a compound produced during chlorophyll degradation [24].

**References**

1. Huneck S, Yoshimura I. Identification of lichen substances. Berlin: Springer Berlin Heidelberg; 1996. 493 p.

2. Huneck S, Feige GB, Schmidt J. Chemie von *Cladonia furcata* und *Cladonia rangiformis*. Herzogia 2004; 17: 51–58.

3. Yoshimura I, Kinoshita Y, Yamamoto Y, Huneck S, Yamada Y. Analysis of secondary metabolites from lichen by high performance liquid chromatography with a photodiode array detector. Phytochem Analysis 1994; 5: 197–205.

4. Huneck S, Benno G, Lumbsch HT. High performance liquid chromatographic analysis of aliphatic lichen acids. Phytochem Analysis 1994; 5: 57–60.

5. Huovinen K, Ahti T, Stenroos S. The composition and content of aromatic lichen substances in *Cladonia* section *Cladonia* and group *Furcatae*. Bibl lichenol. 1990; 38: 209–241.

6. Huovinen K. Variation of lichen acids in *Cladina stellaris* and *Cladina rangiferina* in Finland and northern Norway. Acta Pharm Fenn. 1985; 94: 113–123.

7. Huovinen K, Ahti T. The composition and contents of aromatic lichen substances in the genus *Cladina*. Acta Bot Fenn. 1986; 23: 93–106.

8. Hyvärinen M, Walter B, Koopmann R. (econdary metabolites in *Cladina stellaris* in relation to reindeer grazing and thallus nutrient content. Oikos 2002; 96: 273–280.

9. Stark S, Hyvärinen M. Are phenolics leaching from the lichen *Cladina stellaris* sources of energy rather than allelopathic agents for soil microorganisms?. Soil Biol Biochem. 2003; 35: 1381–1385.

10. BeGora MD, Fahselt D. Usnic acid and atranorin concentrations in lichens in relation to bands of UV irradiance. Bryologist 2001; 104: 134–140.

11. Hall RSB, Bornman JF, Björn LO. UV-induced changes in pigment content and light penetration in the fruticose lichen *Cladonia arbuscula* ssp. *mitis*. J Photoch Photobio B: Biology 2002; 66: 13–20.

12. Mirando M, Fahselt D. The effect of thallus age and drying procedure on extractable lichen substances. Can J Bot. 1978; 56: 1499–1504.

13. Malicki J. The effect of lichen acids on the soil microorganisms. Part I. The washing down of the acids into the soil. Annales Universitatis Mariae Curie-Sklodowska Lublin, Polonia 1965; 10: 239–248.

14. Díaz EM, Armas R, de Oliveira AK, Vicente C, Santiago R, Pereira EC, et al. Effect of soil moisture on the percolation of lichen substances from *Cladonia verticillaris* (Raddi) Fr. in quarzarenic neosol from Brazil. IAL 8, Helsinki, 2016; p 109.

15. Yamamoto Y. Watanabe A. Fatty acid composition of lichens and their phyco- and mycobionts. J Gen Appl Microbiol. 1994; 86: 83–86.

16. Dembitsky VM, Rezanka T, Bychek IA, Shustov MV. Identification of fatty acids from *Cladonia* lichens. Phytochemistry 1991; 30: 4015–4018.

17. Řezanka T, Dembitsky VM. Fatty acids of lichen species from Tian Shan Mountains. Folia Microbiol. 1999; 44: 643–646.

18. Vu TH, Catheline D, Delmail D, Boustie J, Legrand P, Lohézic-Le Dévéhat F. Gas chromatographic analysis to compare the fatty acid composition of fifteen lichen species, with a focus on *Stereocaulon*. Lichenologist 2016; 48: 323–337.

19. Manocha MS, Deven JM. Host-Parasite relations in a mycoparasite IV. A correlation between the levels of γ-linolenic acid and parasitism of *Piptocephalis virginiana*. Mycologia 1975; 67: 1148–1157.

20. Madi L, Wang XJ, Kobiler A, Lichter A, Prusky D. Stress on avocado fruits regulates delta(9)-stearoyl ACP desaturase expression, fatty acid composition, antifungal diene level and resistance to Colletotrichum gloeosporioides attack. Physiol Mol Plant Pathol. 2003; 62: 277–283.

21. Medeira C, Quartin V, Maia I, Diniz I, Matos MC, Semedo JN, et al. Cryptogein and capsicein promote defence responses in *Quercus suber* against *Phytophthora cinnamomi* infection. Eur J Plant Pathol 2012; 134: 145–159.

22. Walley JW, Kliebenstein DJ, Bostock RM, Dehesh K. Fatty acids and early detection of pathogens. Curr Opin Plant Biol. 2013; 16: 520–526.

23. Bourgeois G, Suire C, Vivas N, Vitry C. Atraric acid, a marker for epiphytic lichens in the wood used in cooperage: identification and quantification by GC/MS/(MS). Analusis 1999; 27: 281–283.

24. Matile P, Hörtensteiner S, Thomas H. Chlorophyll degradation. Annu Rev Plant Biol. 1999; 50: 67–95.

**Table 1. Compounds identified in *Cladonia rangiformis* using HPLC and UPLC-MS (mean ± SD; N=2).**

| **Compound** | **R_t (min)_** | **UV (nm) λ_max_** | **M-1** | **mg/g d.w.** | **ESI- (m/z) (rel. int. %)** | **Reference** |
| --- | --- | --- | --- | --- | --- | --- |
|  |  |  |  |  |  |  |
| 1. Protocetraric acid^1, 2^ | 22.3/4.59 | 242, 315 | 373 | 0.839 ± 0.704 | 373 (100), 355 (11), 329 (30), 311(35), 285 (30), 267 (10), 257 (9), 255 (21), 239 (1), 229 (12), 213 (10), 151 (5) | Yoshimura et al. 1994; Huneck et al. 2004 |
| 2. Fumarprotocetraric acid ^1, 2^ | 24.9/5.07 | 210, 239, 318 | 471 | 10.046 ± 12.328 | 471 (7), 355 (75), 311 (100) | Yoshimura et al. 1994; Huneck et al. 2004 |
|  |  |  |  |  |  |  |
| 3. Atranorin ^1,2^ | 33.4/10.56 | 210, 252, 320 | 373 | 21.099 ± 2.975 | 373(100), 341(10), 195(95), 177(60), 75(75) | Yoshimura et al. 1994; Huneck et al. 2004 |
|  |  |  |  |  |  |  |
| 4. 1-methyl 3,4-dicarboxy-hexadecanoate ^2^ | ̶ /10.89 | — | 357 | — | 357 (95), 325 (100), 281 (21), 265 (5), 237 (27), 227 (0.5) | Huneck et al. 2004 |
|  |  |  |  |  |  |  |
| 5. Nor-Rangiformic acid ^2^ | ̶ /11.09 | — | 371 | — | 371(50), 353(100), 309(5), 293(2), 265(10), 255 | Huneck et al. 2004 |
|  |  |  |  |  |  |  |
| 6. Rangiformic acid ^2^ | ̶ /12.03 | — | 385 | — | 385(100), 353(63), 309(10), 293(2), 265(13), 255 | Huneck et al. 2004 |
|  |  |  |  |  |  |  |
| ***Not identified*** |  |  |  |  |  |  |
|  |  |  |  |  |  |  |
| 7. unknown ^1, 2^ | 20.50/4.49 | 239, 259, 282, 340 | 385 | 1.033 ± 0.307 | 385(100), 357(42), 233(30), 195(30), 171(15), 138(10) | This study |
|  |  |  |  |  |  |  |
| 8. unknown ^1, 2^ | 27.29/7.31 | 271, 312 | 517 | — | 517(100), 401(60), 389(18), 179(90) | This study |
|  |  |  |  |  |  |  |
| 9. unknown ^1, 2^ | 29.85/8.93 | 250, 320 | 505 | 0.879 ± 0.509 | 505(62), 385(40), 359(100), 339(33), 233(48), 177(60), 149(60) | This study |
|  |  |  |  |  |  |  |
| 10. unknown ^2^ | ̶ /5.43 | 273 | 417 | — | 417(100), 387(˂10), 341(˂10) | Huneck et al. 2004 |
|  |  |  |  |  |  |  |
| 11. unknown ^2^ | ̶ /5.50 | 273 | 417 | — | 417(100), 387(˂10), 341(˂10) | Huneck et al. 2004 |
|  |  |  |  |  |  |  |
| 12. unknown ^2^ | ̶ /6.38 | 244, 310 | 357 | — | 357(100), 311(91) | Huneck et al. 2004 |
|  |  |  |  |  |  |  |
| 13. unknown ^2^ | ̶ /7.79 | 250-270 | 385 | — | 385(100), 353(10), 339(25) | Huneck et al. 2004 |
|  |  |  |  |  |  |  |
| 14. unknown ^2^ | ̶ /9.29 | 240-280 | 385 | — | 385(100), 353(10), 339(20), | Huneck et al. 2004 |

1. Compound found in HPLC; 2. Compound found in LC-MS; R_t_ , retention time of each compound in HPLC/in UPLC-MS in ESI mode.

**Table 2. Identification and quantification of fatty acids and other compounds in *Cladonia rangiformis* by GC-MS (mean ± SD; N=2).**

| **Fatty acid** | **R_t (min)_** | **mg/g dry weight** |
| --- | --- | --- |
| **FAMEs** |  |  |
| 1. Saturated |  | 0.672 ± 0.402 |
| Lauric acid (12:0) | 5.56 | 0.029 ± 0.021 |
| Myristic acid (14:0) | 7.84 | 0.021 ± 0.010 |
| Pentadecanoic acid (15:0) | 8.98 | 0.014 ± 0.005 |
| Palmitic acid (16:0) | 10.09 | 0.271 ± 0.177 |
| Stearic acid (18:0) | 12.15 | 0.223 ± 0.125 |
| Nonadecanoic acid (19:0) | 13.17 | 0.030 ± 0.015 |
| Arachidic acid (20:0) | 14.30 | 0.024 ± 0.018 |
| Behenic acid (22:0) | 17.02 | 0.032 ± 0.016 |
| Lignoceric acid (24:0) | 21.72 | 0.027 ± 0.014 |
| 2. Monounsaturated |  | 0.417 ± 0.275 |
| Palmitoleic acid (16:1n-7) | 10.37 | 0.029 ± 0.012 |
| Oleic acid (18:1n-9) | 12.36 | 0.282 ± 0.187 |
| Vaccenic acid (18:1n-7) | 12.43 | 0.106 ± 0.116 |
| 3. Polyunsaturated n-6 |  | 1.366 ± 0.547 |
| Linoleic acid (18:2n-6) | 12.84 | 1.321 ± 0.550 |
| Eicosadienoic acid (20:2n-6) | 15.20 | 0.045 ± 0.018 |
| 4. Polyunsaturated n-3 |  | 0.221 ± 0.128 |
| Hexadecatetraenoic acid (16:4n-3) |  | 0.058 ± 0.025 |
| Alpha-linolenic acid (18:3n-3) | 13.530 | 0.163 ± 0.105 |
| **FFAs** |  | 0.290 ± 0.230 |
| 1. Saturated |  |  |
| Palmitic acid (16:0) | 18.16 | 0.090 ± 0.075 |
| 2. Polyunsaturated n-6 |  |  |
| Linoleic acid (18:2n-6) | 26.09 | 0.200 ± 0.179 |
| **TOTAL FA** |  | 3.456 ± 1.330 |
| **Other substances** |  |  |
| Atraric acid | 23.38 | — |
| Phytol | 14.01 | — |
| o-Orcellinaldehyde | 25.50 | — |

R_t_ retention time; esterified fatty acids (FAME), free fatty acids (FFA), fatty acids (FA)
