# Supplementary material for: Genetic variation and factors affecting the genetic structure of the lichenicolous fungus Heterocephalacria bachmannii (Filobasidiales, Basidiomycota)
Source: PLoS One. 2017 Dec 18;12(12):e0189603. doi: 10.1371/journal.pone.0189603 (PMC5734755; doi:10.1371/journal.pone.0189603)

S1 Fig. Phylogenetic tree of ML analysis based on ITS rDNA and LSU rDNA. Bootstrap values ≥ 75% are indicated on the branches.
The clade with the samples of *H. bachmannii* is indicated on gray.

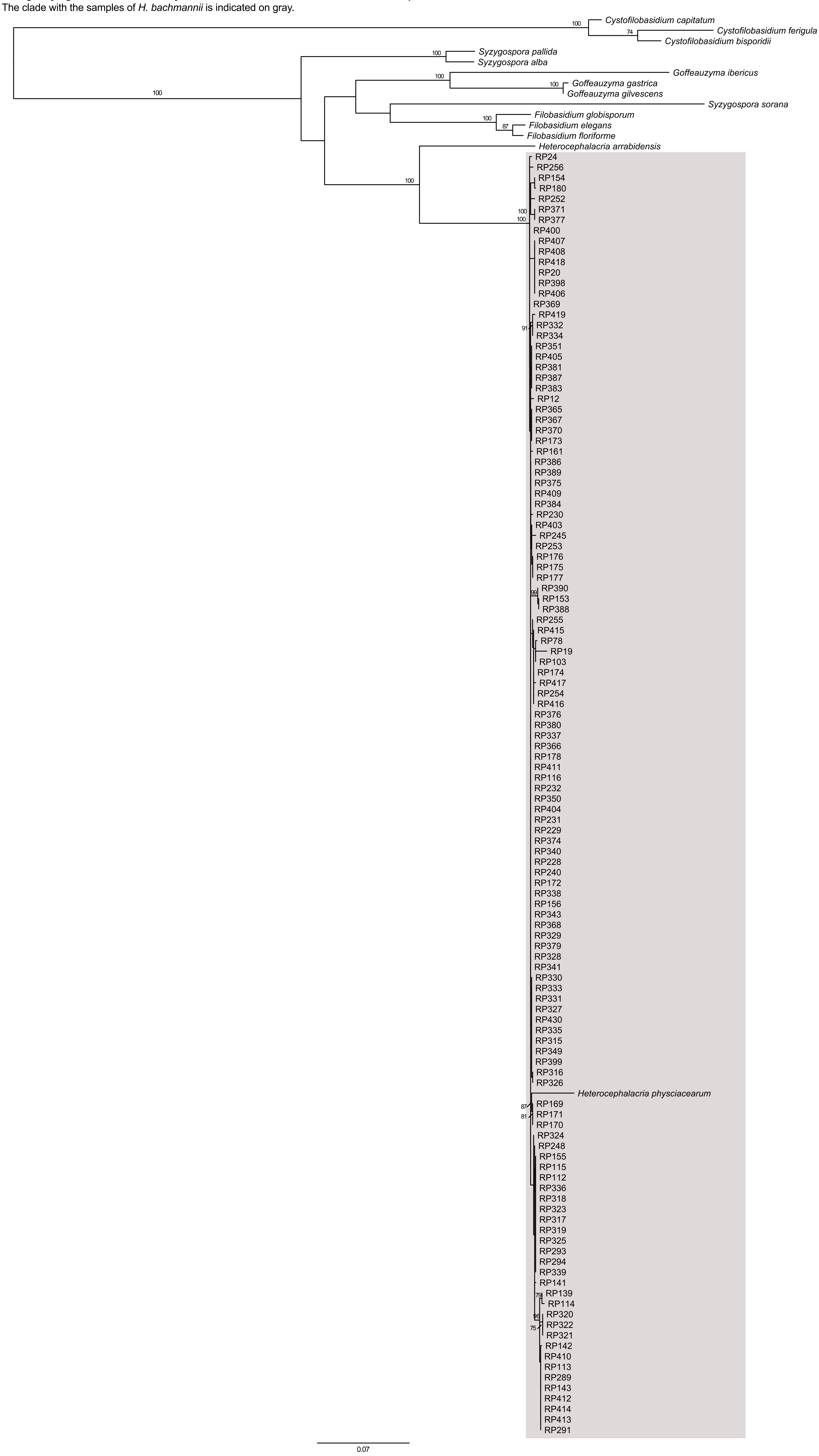

Supplement: S1 Fig — (PDF) [file pone.0189603.s002.pdf]
