# Supplementary material for: Genetic variation and factors affecting the genetic structure of the lichenicolous fungus Heterocephalacria bachmannii (Filobasidiales, Basidiomycota)
Source: PLoS One. 2017 Dec 18;12(12):e0189603. doi: 10.1371/journal.pone.0189603 (PMC5734755; doi:10.1371/journal.pone.0189603)
Supplement: S1 Table — (DOC) [file pone.0189603.s003.doc]

**S1 Table. Sequences downloaded from GenBank to assess the monophyly of *H. bachmannii.***

| **Taxa** | **ID** | **ITS rDNA** | **LSU rDNA** |
| --- | --- | --- | --- |
| *Cystofilobasidium bisporidii* | CBS6346 | KF036597 | AF189832 |
| *Cystofilobasidium capitatum* | YHRM57 | KM384145 | - |
| *Cystofilobasidium ferigula* | CBS7202 | KF036598 | KM079159 |
| *Filobasidium elegans* | CBS 13671 | KP346977 | KP346935 |
| *Filobasidium floriforme* | CBS6241 | AF190007 | AF075498 |
| *Filobasidium globisporum* | CBS 7642 | NR_119453 | AF075495 |
| *Goffeauzyma gastrica* | T23Cga | JQ857024 | JQ857000 |
| *Goffeauzyma gilvescens* | T17Cgi | JQ857030 | JQ857006 |
| *Goffeauzyma ibericus* | CBS 10871 | KF036592 | AY731791 |
| *Heterocephalacria arrabidensis* | CBS 8678 | AF444362 | AF181535 |
| *Heterocephalacria physciacearum* | AM17 | JN053507 | JN043614 |
| *Piskurozyma sorana* | UBC F16310 | EU541305 | EU541305 |
| *Syzygospora alba* | AM147 | JN053509 | JN043616 |
| *Syzygospora pallida* | AM26 | JN053508 | JN043615 |
