# Supplementary material for: Genetic variation and factors affecting the genetic structure of the lichenicolous fungus Heterocephalacria bachmannii (Filobasidiales, Basidiomycota)
Source: PLoS One. 2017 Dec 18;12(12):e0189603. doi: 10.1371/journal.pone.0189603 (PMC5734755; doi:10.1371/journal.pone.0189603)
Supplement: S2 Table — All the specimens had membership coefficients ≥ 0.7 and they were assigned to cluster 1 or 2 without uncertainty. Chemotypes, host species and geographical origin. (DOC) [file pone.0189603.s004.doc]

**S2 Table. Assignment of *H. bachmannii* specimens to the clusters infered in STRUCTURE**. All the specimens had membership coefficients ≥ 0.7 and they were assigned to cluster 1 or 2 without uncertainty.

|  | Cluster 1 (N = 28 ) | Cluster 2 (N = 84) |
| --- | --- | --- |
| **Chemotype** |  |  |
| ATR, RANG, NRANG | 11 | 0 |
| ATR, RANG, NRANG, FUM, PRO | 15 | 5 |
| FUM, PRO | 2 | 49 |
| SQUA | 0 | 5 |
| USN, SQUA | 0 | 6 |
| THAM | 0 | 1 |
| USN, FUM, PRO | 0 | 5 |
| FUM, PRO, BOU | 0 | 10 |
| THAM, BAR | 0 | 2 |
| ATR, FUM, PRO | 0 | 1 |
| **Geographical region** |  |  |
| Southern Europe | 17 | 32 |
| The Azores | 10 | 21 |
| Southern Finland | 0 | 23 |
| Asia | 1 | 1 |
| America | 0 | 8 |
| **Host species** |  |  |
| *C. cervicornis* | 1 | 0 |
| *C. coniocraea* | 0 | 6 |
| *C. cornuta* | 0 | 1 |
| *C. crispata* | 0 | 2 |
| *C. foliacea* | 0 | 3 |
| *C. furcata* | 1 | 16 |
| *C. glauca* | 0 | 3 |
| *C. gracilis* | 0 | 11 |
| *C. granulosa* | 0 | 1 |
| *C. macilenta* | 0 | 0 |
| *C. macroceras* | 0 | 1 |
| *C. mitis* | 0 | 2 |
| *C. phylophora* | 0 | 1 |
| *C. pyxidata* | 0 | 5 |
| *C. ramulosa* | 0 | 7 |
| *C. rangiformis* | 26 | 5 |
| *C. squamosa* | 0 | 2 |
| *C. stereoclada* | 0 | 10 |
| *C. stygia* | 0 | 1 |
| *C. uncialis* | 0 | 6 |

ATR = atranorin, FUM = fumarprotocetraric acid, PRO = Protocetraric acid, RANG = rangiformic acid, NRANG = nor-rangiformic acid.
