# Supplementary material for: Genetic variation and factors affecting the genetic structure of the lichenicolous fungus Heterocephalacria bachmannii (Filobasidiales, Basidiomycota)
Source: PLoS One. 2017 Dec 18;12(12):e0189603. doi: 10.1371/journal.pone.0189603 (PMC5734755; doi:10.1371/journal.pone.0189603)
Supplement: S3 Table — (DOC) [file pone.0189603.s005.doc]

**S3 Table. Assignment of *H. bachmannii* specimens (on *C. rangiformis*) to the clusters inferred in Structure. All the specimens had membership coefficients ≥ 0.79 and they were assigned to cluster 1 or 2 without uncertainty.**

|  | Cluster 1 (N = 13 ) | Cluster 2 (N = 18) |
| --- | --- | --- |
| **Chemotype** |  |  |
| ATR, RANG, NRANG | 5 | 9 |
| ATR, RANG, NRANG, FUM, PRO | 8 | 9 |
| **Geographical region** |  |  |
| Southern Europe | 6 | 9 |
| The Azores | 7 | 7 |
| Southetn Finland | 0 | 2 |

ATR = atranorin, FUM = fumarprotocetraric acid, PRO = Protocetraric acid, RANG = rangiformic acid, NRANG = nor-rangiformic acid.
