# Supplementary material for: Genetic variation and factors affecting the genetic structure of the lichenicolous fungus Heterocephalacria bachmannii (Filobasidiales, Basidiomycota)
Source: PLoS One. 2017 Dec 18;12(12):e0189603. doi: 10.1371/journal.pone.0189603 (PMC5734755; doi:10.1371/journal.pone.0189603)
Supplement: S4 Table — (DOCX) [file pone.0189603.s006.docx]

S4 Table. List of primers used in PCR and sequencing reactions

| **Locus** | **Primer** | **Sequence (5’-3’)** | **Organism** |
| --- | --- | --- | --- |
| ITS rDNA | ITS1F | CTTGGTCATTTAGAGGAAGTAA | Host & parasite |
|  | ITS4 | TCCTCCGCTTATTGATATGC | Host |
|  | BasidLSU3-3 | GACTGACTTCAATCGT | Parasite |
| LSU rDNA | LROR | ACCCGCTGAACTTAAGC | Host |
|  | LR5 | TCCTGAGGGAAACTTCG | Host & parasite |
|  | BasidLSU3-5 | ACGATTGAAGTCAGTCG | Parasite |
| mtSSU | MS1 | CAGCAGTCAAGAATATTAGTCAATG | Parasite |
|  | MS2 | GCGGATTATCGAATTAAATAAC | Parasite |
| IGS rDNA | IGSf | AGTGGCCGWTRGCTATCATT | Host |
|  | IGSr | AATTGCAGCAATTGCTCG | Host |
|  | LR12R | CTGAACGCCTCTAAGTCAGAA | Parasite |
|  | 5SRNA | ATCAGACGGGATGCGGT | Parasite |
| l41 | L41F | GGTCAACGTTCCCAAGACTC | Parasite |
|  | L41R | CCCTTCTTGTACTGGGTCAC | Parasite |
| RPB2 | bRPB2-6F | TGGGGYATGGTNTGYCCYGC | Parasite |
|  | bRPB2-11R | CAATCWCGYTCCATYTCWCC | Parasite |
| RPB1 | RPB1-Af | GARTGYCCDGGDCAYTTYGG | Parasite |
|  | RPB1-CR | CCNGCDATNTCRTTRTCCATRTA | Parasite |
| EF1α | 983F | GCYCCYGGHCAYCGTGAYTTYAT | Parasite |
|  | 1567 | ACHGTRCCRATACCACCRATCTT | Parasite |
| ATP6 | ATP6-1 | ATTAATTSWCCWTTAGAWCAATT | Parasite |
|  | ATP6-2 | TAATTCTANWGCATCTTTAATRTA | Parasite |
